# ^19^F MRI-fluorescence imaging dual-modal cell tracking with partially fluorinated nanoemulsions

**DOI:** 10.3389/fbioe.2022.1049750

**Published:** 2022-11-03

**Authors:** Ting Tang, Qiang Zhu, Shuang Liu, Hailong Dai, Yu Li, Caihong Tang, Kexin Chen, Mou Jiang, Lijun Zhu, Xin Zhou, ShiZhen Chen, Zitong Zheng, Zhong-Xing Jiang

**Affiliations:** ^1^ Hunan Provincial Key Laboratory of Tumor Microenvironment Responsive Drug Research, Department of Pharmacy, Hengyang Medicinal School, The Second Affiliated Hospital, University of South China, Hengyang, Hunan, China; ^2^ State Key Laboratory of Magnetic Resonance and Atomic and Molecular Physics, National Center for Magnetic Resonance in Wuhan, Wuhan Institute of Physics and Mathematics, Innovation Academy for Precision Measurement Science and Technology, Chinese Academy of Sciences, Wuhan, China; ^3^ Department of Pediatrics, Affiliated Hospital of Changchun University of Traditional Chinese Medicine, Changchun, China

**Keywords:** ^19^F MRI, fluorescence, cell tracking, partially fluorinated, nanoemulsions

## Abstract

As a noninvasive “hot-spot” imaging technology, fluorine-19 magnetic resonance imaging (^19^F MRI) has been extensively used in cell tracking. However, the peculiar physicochemical properties of perfluorocarbons (PFCs), the most commonly used ^19^F MRI agents, sometimes cause low sensitivity, poor cell uptake, and misleading results. In this study, a partially fluorinated agent, perfluoro-*tert*-butyl benzyl ether, was used to formulate a ^19^F MRI-fluorescence imaging (FLI) dual-modal nanoemulsion for cell tracking. Compared with PFCs, the partially fluorinated agent showed considerably improved physicochemical properties, such as lower density, shorter longitudinal relaxation times, and higher solubility to fluorophores, while maintaining high ^19^F MRI sensitivity. After being formulated into stable, monodisperse, and paramagnetic Fe^3+^-promoted nanoemulsions, the partially fluorinated agent was used in ^19^F MRI-FLI dual imaging tracking of lung cancer A549 cells and macrophages in an inflammation mouse model.

## Introduction

Tracking cells *in vivo* with imaging technologies is highly important for biology, pathology, and medicine (Hong et al., 2011; Kircher et al., 2011; Bulte and Daldrup-Link, 2018). Such imaging technologies may provide real-time, noninvasive, quantitative, and multidimensional information about the cells for a better understanding of the biological and pathological processes on a cellular level, thus promoting accurate diagnosis and effective therapy. For example, tracking the circulating cancer cells *in vivo* with imaging technology may shed light on cancer metastasis mechanisms and therapeutic strategies (Xia et al., 2012; Nedosekin et al., 2013; Lee et al., 2018; Dutta et al., 2019; Deán-Ben et al., 2020), while monitoring macrophages may help visualize the immune response to inflammation (Stoll et al., 2012; Bouvain et al., 2019).

In recent years, considerable attention has been given to developing novel imaging technologies for cell tracking. Among the imaging technologies, fluorescent protein-based fluorescence imaging (FLI) (Chudakov et al., 2003; Sun et al., 2009; Ghosh et al., 2019), fluorescence dye-based FLI (Bulte et al., 2004; Maška et al., 2013; Tian et al., 2020), paramagnetic nanoparticle-based ^1^H MRI (Cromer Berman et al., 2011; Ahrens and Zhong, 2013; Li et al., 2013), and fluorinated nanoemulsion-based ^19^F MRI (Ferreira et al., 2008; Bulte, 2009; Wang et al., 2013) are among the most used ones. Since each imaging technology has its strengths and weaknesses, tracking cells with multimodal imaging is highly preferred, which takes advantage of each imaging technology and provides accurate, detailed, and multidimensional information. During cell tracking, the cells are usually labeled *in vitro* and tracked *in vivo*, and some special cells, such as macrophages, may also be labeled and tracked *in vivo*. FLI is advantageous for *in vitro* cellular study due to its convenience, high sensitivity, and resolution. Meanwhile, ^19^F MRI perfectly overcomes the tissue-depth limit of FLI during the *in vivo* animal study and provides noninvasive, quantitative, and background-free “hot-spot” cell images without ionizing radiation. Thus, ^19^F MRI-FLI dual imaging systems have shown significant potential for clinical application.

Although some ^19^F MRI-FLI dual imaging systems have recently been developed for cell tracking (Ahrens et al., 2005; Gaudet et al., 2017; Peng et al., 2018), many issues remain unaddressed. On the one hand, the low sensitivity of ^19^F MRI requires high ^19^F MRI agent-loading per cell and a large number of ^19^F-labeled cells to achieve clear cell images (Ahrens et al., 2005; Gaudet et al., 2017; Peng et al., 2018). Furthermore, the non-symmetric allocation of fluorine atoms in most perfluorocarbon (PFC)-based imaging agents leads to severe signal splitting, low ^19^F MRI sensitivity, and chemical shift imaging artifacts. On the other hand, the peculiar physicochemical properties of PFCs, such as high hydrophobicity, low polarity, and low solubility in hydrocarbons, usually result in complex formulation and severe internal organ retention (Meyer et al., 1992; Kimura et al., 2004; Janjic et al., 2008). Notably, the extremely low solubility and interaction of fluorescent dyes in PFCs may cause aggregation-induced quenching (ACQ) of fluorescence, difficulties in encapsulation, and early release of fluorescent dyes (Würthner et al., 2011; Battistelli et al., 2016). Moreover, the high density of PFCs and their nanoemulsions may cause low cell uptake, cell damage, and misleading results (Patel et al., 2013). To address these issues, we recently used partially fluorinated agents, hydrofluorocarbons (HFCs), as alternatives for PFCs (Chen et al., 2022; Wu et al., 2022). These commercially available HFCs had a unified ^19^F NMR peak, considerable solubility toward fluorescent dyes and functional agents, relatively low density and high ^19^F MRI sensitivity, and efficient cell uptake. However, significant cytotoxicity was found in the HFC nanoemulsions, which promoted the synthesis of highly sensitive and biocompatible HFC agents.

**FIGURE 1:**
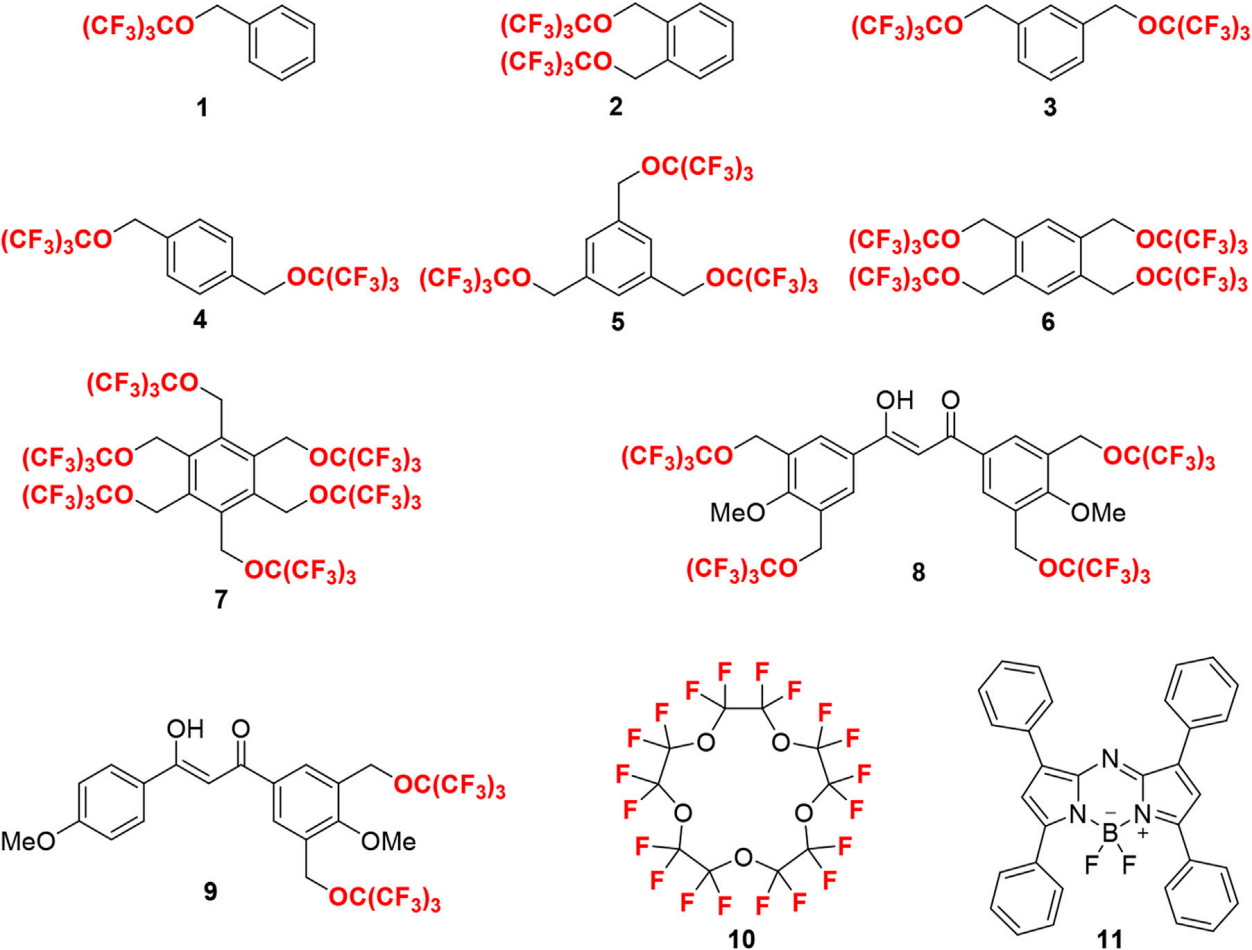
Structures of HFCs 1–7, chelators 8 and 9, perfluoro-15-crown-5 10, and aza-BODIPY 11.

Herein, we have developed novel partially fluorinated nanoemulsions for efficient ^19^F MRI cell tracking through synthesizing, screening, and formulating a series of HFCs. Because of their high ^19^F MRI sensitivity, biocompatibility, and stability (Wu et al., 2021), perfluoro-*tert*-butyl benzyl ethers 1–7 were designed as HFCs (Figure 1). Three strategies have been used to improve the ^19^F MRI sensitivity. First, 1–6 symmetrical PFTB groups were introduced into the benzyl cores to generate an intense unified singlet ^19^F NMR peak from 9 to 54 equivalent ^19^F atoms in HFCs 1–7, that is, the utility of every ^19^F atom for sensitive ^19^F MRI. Second, the π–π interactions among the benzene cores of HFCs 1–7 may slow their movements and thus shorten the longitudinal relaxation time (T_1_), which would shorten the data collection time during ^19^F MRI and thus improve the signal-to-noise ratio per unit time for sensitive ^19^F MRI. Third, perfluoro-*tert*-butoxylated diketone chelators 8 and 9 may capture paramagnetic ions into the HFCs and further shorten T_1_ through the paramagnetic relaxation enhancement (PRE) effect for highly sensitive ^19^F MRI. Due to their similar hydrofluorocarbon structures, chelators 8 and 9 may be highly soluble in HFCs 1–7 and provide a unified ^19^F NMR peak from the mixture. In these ways, a unified ^19^F NMR peak with an ultrashort T_1_ would be generated for highly sensitive ^19^F MRI cell tracking. The near-infrared fluorescence of the resulting fluorinated nanoemulsion may be realized by aza-BODIPY 11. Compared to highly fluorinated PFCs, HFCs 1–7 with a benzene core and lower fluorine content may have much higher solubility toward aza-BODIPY 11 and thus improve the FLI capability. Moreover, the co-solubility and improved interactions among HFCs 1–7, chelators 8 and 9, and aza-BODIPY 11 may stabilize the nanoemulsion for high biocompatibility. Perfluoro-15-crown-5 10, a widely used PFC in ^19^F MRI, was employed as a control to illustrate the impact of π–π interactions and enhanced solubility.

## Materials and methods

### General information

^1^H, ^13^C, and ^19^F NMR spectra were recorded on a Bruker 400 MHz or 500 MHz. Chemical shifts and coupling constants (*J*) were provided in ppm and Hertz (Hz), respectively. ^1^H NMR spectra were referenced to tetramethylsilane (d, 0.00 ppm) using CDCl_3_ (s, 7.26 ppm) or acetone-d_6_ (m, 2.05 ppm) as the solvent. ^13^C NMR spectra were referenced to solvent carbons (77.2 ppm for CDCl_3_ and 29.8 ppm for acetone-d_6_). ^19^F NMR spectra were referenced to 2% hexafluorobenzene (s, −164.90 ppm) in CDCl_3_. The splitting patterns for ^1^H NMR and ^19^F NMR spectra were denoted as follows: s = singlet, d = doublet, t = triplet, q = quartet, and m = multiplet.

Phospholipid S75 was purchased from Lipoid GmbH (Ludwigshafen, Germany). Pluronic F68 (average MW = 8,350) was obtained from Adamas (Shanghai, China). Medicinal-grade soybean oil was acquired from Aladdin (Shanghai, China). Aza-BODIPY was synthesized in this lab according to the method used by Jokic et al. (2012). Human breast cancer MCF-7 cells and human triple-negative breast cancer MDA-MB-231 cells were purchased from the Cell Bank of the Chinese Academy of Sciences (Shanghai, China). The human lung adenocarcinoma cell line A549 was purchased from Beyotime (Shanghai, China). BALB/c mice (female, 5–6 weeks old) and BALB/c nude mice (female, 5–6 weeks old) were purchased from Hubei BIONT Biotechnology Co., Ltd. Unless otherwise indicated, all reagents were obtained from a commercial supplier and used without prior purification. All solvents were either analytical or HPLC grade. Deionized water was used unless otherwise indicated. THF was dried and freshly distilled before use. Column flash chromatography was performed on silica gel (200–300 mesh) with the eluent as indicated in the procedures. All animal studies were conducted according to the experimental practices and standards approved by the Animal Welfare and Research Ethics Committee at the Innovation Academy for Precision Measurement Science and Technology, Chinese Academy of Sciences.

The size distribution and polymer dispersion index (PDI) of nanoparticles were determined by a dynamic light-scattering (DLS) instrument (Malvern, Nano ZS 90, United Kingdom). UV-Vis and fluorescence emission spectra were obtained using a UV-2600 UV-Vis spectrophotometer (Shimadzu, Japan) and a FluoroMax-4 spectrofluorometer (HORIBA Scientific, America), respectively. Aza-BODIPY encapsulation efficiency (EE%) and drug loading efficiency (DLE%) were determined using an LC20-AT reversed-phase high-performance liquid chromatograph (Shimadzu, Japan). *In vitro* cellular uptake was determined by a Leica-TCS-SP8-STED CLMS (Leica, Germany). Cell viability (%) was determined using an ELx800 light-absorption microplate reader (BioTek, America). *In vivo* fluorescence imaging was determined using an IVIS Spectrum *in vivo* imaging system (PerkinElmer, America).

### Synthesis of HFCs 1–7 and chelators 8 and 9

HFC 1: Under an argon atmosphere, a solution of potassium perfluoro-*tert*-butoxide (15.0 g, 54.7 mmol) in anhydrous *N,N*-dimethylformamide (DMF, 100 ml) was added to a reaction flask containing benzyl bromide 12 (7.8 ml, 65.6 mmol). The reaction mixture was stirred at 40°C for 12 h until thin-layer chromatography (TLC) analysis showed that benzyl bromide was consumed completely. Then, the reaction mixture was quenched with water, and the lower phase was collected as a yellowish oil, which was washed with water thrice to give HFC 1 as a colorless oil (15.9 g, 89% yield). ^1^H NMR (400 MHz, CDCl_3_) δ 7.39–7.31 (m, 5H) and 5.02 (s, 2H). ^19^F NMR (376 MHz, CDCl_3_) δ −73.11 (s).

General synthetic procedures for HFCs 2–6 using the synthesis of HFC 2 as an example: Under an argon atmosphere, a solution of potassium perfluoro-*tert*-butoxide (6.2 g, 22.6 mmol) in anhydrous DMF (25 ml) was added to a reaction flask containing bromide 13 (2.0 g, 7.6 mmol). The reaction mixture was stirred at room temperature for 24 h until TLC showed that the starting material was consumed completely. The reaction mixture was quenched with water, and the white precipitate was collected and washed with water several times to give HFC 2 (Zhao et al., 2012) as a white powder (3.0 g, 69% yield). ^1^H NMR (400 MHz, CDCl_3_) δ 7.43–7.39 (m, 1H), 7.34–7.32 (m, 3H), and 5.05 (s, 4H).^19^F NMR (376 MHz, CDCl_3_) δ −73.27 (s).

HFC 3 (Zhao et al., 2012) was prepared as a white powder (5.5 g, 84% yield) from 14 (3.0 g, 11.4 mmol) using the same procedure as that for compound 2, with an increased amount of potassium perfluoro-*tert*-butoxide (9.3 g, 33.9 mmol). ^1^H NMR (400 MHz, CDCl_3_) δ 7.42 (s, 4H) and 5.12 (s, 4H). ^19^F NMR (376 MHz, CDCl_3_) δ −73.31 (s).

HFC 4 (Zhao et al., 2012) was prepared as a white powder (0.9 g, 83% yield) from 15 (0.5 g, 1.9 mmol) using the same procedure as that for compound 2, with an increased amount of potassium perfluoro-*tert*-butoxide (2.1 g, 7.7 mmol). ^1^H NMR (400 MHz, acetone-d_6_) δ 7.38 (s, 4H) and 5.08 (s, 4H). ^19^F NMR (376 MHz, acetone-d_6_) δ −71.18 (s).

HFC 5 (Zhao et al., 2012) was prepared as a white powder (4.6 g, 89% yield) from 16 (2.0 g, 5.6 mmol) using the same procedure as that for compound 2, with an increased amount of potassium perfluoro-*tert*-butoxide (6.1 g, 22.3 mmol). ^1^H NMR (400 MHz, CDCl_3_) δ 7.32 (s, 3H) and 5.07 (s, 6H). ^19^F NMR (376 MHz, CDCl_3_) δ −73.36 (s).

HFC 6 was prepared as a white powder (1.0 g, 80% yield) from 17 (0.5 g, 1.1 mmol) using the same procedure as that for compound 2, with an increased amount of potassium perfluoro-*tert*-butoxide (1.8 g, 6.6 mmol). ^1^H NMR (400 MHz, acetone-d_6_) δ 7.67 (s, 2H) and 5.28 (s, 8H). ^19^F NMR (376 MHz, acetone-d_6_) δ −71.24. ^13^C NMR (101 MHz, acetone-d_6_) δ 135.3, 129.9, 121.3 (q, *J* = 292.9 Hz), 81.2–80.3 (m), and 69.5. HRMS (ESI) calculated for C_26_H_10_F_36_O_4_K^+^ ([M + K]^+^) 1108.9636 found 1108.9628.

Compound 20: Under an argon atmosphere, anhydrous methanol (300 ml) and concentrated sulfuric acid (6.1 ml) were added to a reaction flask containing acid 19 (10.0 g, 55.5 mmol). The resulting mixture was refluxed for 8 h. Then, the reaction was quenched with water and extracted with dichloromethane (DCM, 300 ml, three times). The combined organic layers were dried over anhydrous Na_2_SO_4_ and concentrated under vacuum, and the residue was purified by silica gel column chromatography (PE/EA = 100/1) to give compound 20 (Grimster et al., 2013) as a yellowish oil (11.8 g, 99% yield). ^1^H NMR (400 MHz, CDCl_3_) δ 7.71 (s, 2H), 3.88 (s, 3H), 3.74 (s, 3H), and 2.31 (s, 6H).

Compound 21: Under an argon atmosphere, compound 20 (11.0 g, 56.7 mmol), *N*-bromosuccinimide (NBS, 22.4 g, 125.8 mmol), and azobisisobutyronitrile (AIBN, 58.3 mg, 0.35 mmol) were dissolved in CCl_4_ (230 ml). The resulting mixture was refluxed for 3 h. Then, the reaction was quenched with water and extracted with dichloromethane (DCM, 300 ml, three times). The combined organic layers were dried over anhydrous Na_2_SO_4_ and concentrated under vacuum, and the residue was purified by silica gel column chromatography (PE/EA = 250/1) to give compound 21 as a white powder (11.8 g, 59% yield). ^1^H NMR (400 MHz, CDCl_3_) δ 8.07 (s, 2H), 4.56 (s, 4H), 4.08 (s, 3H), and 3.92 (s, 3H). ^13^C NMR (101 MHz, CDCl_3_) δ 165.7, 160.4, 133. 7, 132.5, 127.0, 62.6 (d, *J* = 1.7 Hz), 52.5 (d, *J* = 1.8 Hz), and 26.9. HRMS (ESI) calculated for C_11_H_12_Br_2_O_3_Na^+^ ([M + Na]^+^ 347.9025 found 347.9020.

Compound 22: Under an argon atmosphere, a solution of potassium perfluoro-*tert*-butoxide (10.9 g, 39.8 mmol) in anhydrous DMF (60 ml) was added to a reaction flask containing compound 21 (4.0 g, 11.4 mmol). The reaction mixture was stirred at 40°C for 12 h. The mixture was quenched with water. The precipitate was collected and washed with water three times. After dissolving in diethyl ether, the precipitate was dried over anhydrous Na_2_SO_4_. Then, the solution was filtered and concentrated to give a residue, which was purified by rapidly flushing the silica gel column with low-boiling petroleum ether to give compound 22 as a white powder (4.7 g, 62% yield). ^1^H NMR (400 MHz, acetone-d_6_) δ 8.04 (s, 2H), 5.18 (s, 4H), 3.82 (s, 3H), and 3.77 (s, 3H). ^19^F NMR (376 MHz, acetone-d_6_) δ −71.01 (s). ^13^C NMR (101 MHz, CDCl_3_) δ 165.9, 161.6, 133.7, 129.0, 127.1, 120.5 (q, *J* = 292.8 Hz), 80.4–79.5 (m), 66.3, 63.8, and 52.6. HRMS (ESI) calculated for C_19_H_12_F_18_O_5_Na^+^ ([M + Na]^+^ 658.0290 found 658.0291.

Compound 24: Under an argon atmosphere, compound 23 (1.0 g, 5.6 mmol, in 20 ml CCl_4_) was added to the reaction flask containing NBS (2.2 g, 12.4 mmol) and AIBN (5.5 mg, 0.033 mmol). After being refluxed at 85°C for 3.5 h, the reaction mixture was quenched with water and extracted with DCM (300 ml, three times). The combined organic layers were dried over anhydrous Na_2_SO_4_, concentrated under vacuum, and purified by silica gel column chromatography (PE/EA = 150/1) to give compound 24 as a white powder (0.96 g, 51% yield). ^1^H NMR (400 MHz, CDCl_3_) δ 7.98 (s, 2H), 4.57 (s, 4H), 4.08 (s, 3H), and 2.60 (s, 3H). ^13^C NMR (101 MHz, CDCl_3_) δ 196.2, 160.6, 134.0, 132.6, 132.5, 62.6, 27.0, and 26.7. HRMS (ESI) calculated for C_11_H_12_Br_2_O_2_Na^+^ ([M + Na]^+^) 358.9076 found 358.9075.

Compound 25: Under an argon atmosphere, a solution of potassium perfluoro-*tert*-butoxide (0.7 g, 2.6 mmol) in anhydrous DMF (15 ml) was added to a reaction flask containing compound 24 (0.3 g, 0.9 mmol). After being stirred at room temperature for 24 h, the reaction mixture was quenched with water. The white precipitate was collected, dissolved in petroleum ether, and dried over anhydrous Na_2_SO_4._ Then, the solution was filtered and concentrated to give a residue, which was purified by rapidly flushing the silica gel column with low-boiling petroleum ether to give compound 25 as a white solid (0.4 g, 72% yield). ^1^H NMR (400 MHz, acetone-d_6_) δ 8.06 (s, 2H), 5.18 (s, 4H), 3.82 (s, 3H), and 2.46 (s, 3H). ^19^F NMR (376 MHz, acetone-d_6_) δ −70.99. ^13^C NMR (101 MHz, CDCl_3_) δ 196.3, 161.4, 134.0, 132.1, 129.2, 120.5 (q, *J* = 293.2 Hz), 80.4–79.3 (m), 66.3, 63.7, and 26.5. HRMS (ESI) calculated for C_19_H_12_F_18_O_4_ Na^+^ ([M + Na]^+^) 669.0340 found 669.0342.

Compound 9: In a glove box, anhydrous tetrahydrofuran (THF, 4 ml) was added to the reaction flask containing compound 22 (441 mg, 0.7 mmol) and *p*-methoxy acetophenone (50 mg, 0.3 mmol). Then, potassium *tert*-butoxide (111 mg, 1.0 mmol) was added. The mixture was stirred at room temperature for 2 h and further stirred at 50°C for 24 h. Subsequently, a 2-N HCl solution was added to neutralize the reaction mixture. After that, the mixture was extracted with diethyl ether (60 ml, three times). The combined organic layers were dried over anhydrous Na_2_SO_4_, concentrated under vacuum, and purified by silica gel column chromatography (PE/EA = 100/1) to give compound 9 as a white solid (55.5 mg, 24% yield). ^1^H NMR (500 MHz, CDCl_3_) δ 8.02–7.94 (m, 4H), 7.00–6.98 (m, 2H), 6.71 (s, 1H), 5.15 (s, 4H), and 3.88 (d, *J* = 3.0 Hz, 6H). ^19^F NMR (376 MHz, CDCl_3_) δ −72.98. ^13^C NMR (101 MHz, CDCl_3_) δ 185.9, 182.9, 163. 6, 160.7, 132.6, 130.7, 129.4, 129.2, 127.9, 120.5 (q, *J* = 293.6 Hz), 114.2, 92.2, 80.5–79.9 (m), 66.4, 63.7, and 55.7. HRMS (ESI) calculated for C_27_H_18_F_18_O_6_Na^+^ ([M + Na]^+^) 803.0708 and found 803.0706.

### Formulation and characterization of partially fluorinated nanoemulsions

The typical procedure for formulating partially fluorinated nanoemulsion using nanoemulsion E1 as an example: The optimized formulation is composed of 36% Lipoid S75, 4.5% soybean oil, 0.5% aza-BODIPY 11, 1% chelator 9, and 58% HFC 1. Briefly, Lipoid S75 and aza-BODIPY 11 were each dissolved in DCM. Chelator 9 (2 mg) was dissolved in HFC 1 (130 mg), and the aza-BODIPY 11 (1 mg, 2.7 mM) solution was added. Then, the solution of Lipoid S75 (80 mg, 20% w/v) was added to the mixture. The resulting mixture was transferred to a 100-ml round-bottom flask and subjected to solvent removal under vacuum at a rotation speed of 50 rpm for 5 min at 37°C, thus forming a thin film. The deionized water (4 ml) was added to the reaction flask under ultrasound. The crude nanoemulsion was sonicated in a water bath for 10 min and in a cell disruptor for 5 min, followed by filtration through a 0.2-µm syringe filter.

Determination of aza-BODIPY encapsulation efficiency (EE%) and drug loading content (DLC%): Different concentrations of aza-BODIPY standard solutions (5 μg/ml, 10 μg/ml, 20 μg/ml, 25 μg/ml, 30 μg/ml, 40 μg/ml, and 50 μg/ml) were prepared with analytical acetonitrile as the solvent, and the standard curve of aza-BODIPY was drawn with high-performance liquid chromatography (HPLC). The chromatographic conditions of HPLC are as follows: column: RP C18 column, 5 μm, 4.6 mm × 100 mm; mobile phase: A as H_2_O and B as CH_3_CN; gradient: B of 70%–100% (30 min); wavelength: 650 nm; injection volume: 20 μl; flow rate: 0.7 ml/min.

Nanoemulsions E1–E4 were centrifuged at 11,000 rpm for 30 min, the concentration of free aza-BODIPY in the supernatant was measured by HPLC, and the mass of free aza-BODIPY (W_f_) was calculated based on the standard curve measured earlier. The total weight of dried nanoemulsions (W_d_) and the total content of aza-BODIPY in nanoemulsions (W_t_) were calculated, and EE% and DLC% were calculated using Equations 1 and 2.

$$EE\%=\left(W_{t}-W_{f}\right)/W_{t}\times 100\%,$$ (1)

$$DLC\%=\left(W_{t}-W_{f}\right)/W_{d}\times 100\%.$$ (2)

### *In vitro* cellular uptake study

A549 cells were cultured in DMEM high-glucose medium with 10% fetal bovine serum and 1% penicillin–streptomycin. All cells were cultured at 37^°^C in a humidified atmosphere containing 5% CO_2_. About 2 × 10^6^ A549 cells were seeded into culture flasks overnight. A culture medium containing nanoemulsions (E1–E4) with 18 mM ^19^F was added. After 12 h incubation at 37^°^C, the medium was removed, and the cells were washed with PBS thrice. Trypsin was added to detach cells, and the cells were resuspended in PBS for counting. Subsequently, the cells were centrifuged, and the supernatant was removed; 400 µl of cell lysis solution was added and then transferred to a 5-mm NMR tube, and a capillary tube containing 100 µl of sodium trifluomethanesulfonate solution was added to measure the content of ^19^F.

About 2 × 10^5^ A549 cells were seeded on 2-cm cell culture dishes for 12 h. A culture medium containing the BODIPY (10 μg/ml)-labeled nanoemulsions (E1–E4) was added. After 12 h of incubation, the medium was removed, and the cells were washed with PBS and fixed with 4% paraformaldehyde for 15 min. Then, fixed cells were stained with 200 µl of 4,6-diamino-2-phenylindole (DAPI) for 10 min and washed with PBS at least three times. Finally, cells were imaged using a confocal laser scanning microscope (CLSM).

### *In vitro* cellular cytotoxicity assay

A549 cells, MCF-7 cells, and MDA-MB-231 cells were cultured in DMEM high-glucose medium with 10% fetal bovine serum and 1% penicillin–streptomycin. All cells were cultured at 37°C in a humidified atmosphere containing 5% CO_2_.

*In vitro* cell cytotoxicity was evaluated using a cell counting (CCK-8) assay. About 1 × 10^4^ cells (A549, MCF-7, and MDA-MB-231, respectively) were seeded per well in 96-well plates (*n* = 3) and cultured for 24 h. Nanoemulsions (E1–E4) were diluted with the medium to a specific concentration and added to each well, respectively. After incubation for 12 h, cells were washed with PBS (pH 7.4) twice. Then, 100 μl of CCK-8 (10% v/v) solution was added to each well and incubated for another 2 h. Finally, the absorbance at 450 nm was measured with a microplate reader.

Cell viability (%) was calculated using the following formula:

$$Cell\;viability\left(\%\right)=\left[\left(A_{Test}-A_{Blank}\right)/\left(A_{Control}-A_{Blank}\right)\right]\times 100\%,$$

where A_Test_, A_Control_, and A_Blank_ represented the absorbance of cells with different treatments, untreated cells, and PBS buffer solution, respectively.

### *In vitro* nanoemulsion ^19^F MRI study

All ^19^F MRI phantom experiments in the *in vitro* nanoemulsion ^19^F MRI study were performed on a 400-MHz Bruker BioSpec MRI system at 25°C. Nanoemulsions E1–E4 were serially diluted with water to give a series of ^19^F concentrations of 40 mM, 20 mM, 10 mM, 5 mM, and 2.5 mM, respectively.

For E1–E4, the ^19^F density-weighted ^19^F MRI phantom images were acquired by using a gradient-echo (GRE) pulse sequence with the following parameters: method = RARE, matrix size = 32 × 32, SI = 20 mm, FOV = 3.0 cm × 3.0 cm, TR = 4,000 ms, TE = 3 ms, NS = 12, and scan time = 384 s. For T_1_-weighted ^19^F MRI phantom images, a gradient-echo (GRE) pulse sequence with the following parameters was used: method = RARE, matrix size = 32 × 32, SI = 20 mm, FOV = 3.0 cm × 3.0 cm, TR = 180 ms, TE = 3 ms, NS = 135, and scan time = 389 s.

### *In vitro* cellular ^19^F MRI study

About 1 × 10^7^ A549 cells were seeded on 10-cm cell culture dishes for 24 h. A culture medium containing nanoemulsions (E1–E4) with 18 mM of ^19^F was added. After 12 h of incubation at 37°C, the medium was removed, and the cells were washed with PBS twice. Trypsin was added to digest the cells, and the cell suspension was transferred into a centrifuge tube. After centrifuging at 2,000 r/min for 5 min, the supernatant was removed, and the cell pellet was resuspended in PBS. The suspension was transferred to a 0.5-ml tube and centrifuged again to pellet the cells. Finally, the 0.5-ml tube was placed in a 10-mm NMR tube for imaging.

All ^19^F MRI phantom experiments in the *in vitro* cellular ^19^F MRI study were performed on a 400-MHz Bruker BioSpec MRI system at 25°C.

The ^19^F density-weighted ^19^F MRI phantom images were acquired by using a gradient-echo (GRE) pulse sequence with the following parameters: method = RARE, matrix size = 32 × 32, SI = 20 mm, FOV = 2.0 cm × 2.0 cm, TR = 4,000 ms, TE = 3 ms, NS = 12, and scan time = 384 s. For T_1_-weighted ^19^F MRI phantom images, a gradient-echo (GRE) pulse sequence with the following parameters was used: method = RARE, matrix size = 32 × 32, SI = 20 mm, FOV = 2.0 cm × 2.0 cm, TR = 180 ms, TE = 3 ms, NS = 135, and scan time = 389 s.

### *In vivo* ^19^F MRI and fluorescence imaging

#### Tracking A549 cancer cells in mice

About 1 × 10^7^ A549 cells were seeded on 10-cm cell culture dishes for 24 h. A culture medium containing nanoemulsions (E1–E2) with 36 mM of ^19^F was added. After 12 h of incubation at 37°C, the medium was removed, and the cells were washed with PBS twice. Trypsin was added to digest the cells; then, the cell suspension was transferred into a centrifuge tube. The suspension was centrifuged at 2,000 r/min for 5 min. The supernatant was discarded, and the pellet was resuspended in PBS; 0.1 ml of PBS (containing about 1 × 107 E1- or E2-labeled cells) was injected subcutaneously into the left and right backs of BALB/c nude mice (*n* = 3), respectively. *In vivo* ^19^F MR images were captured using a 9.4-T nuclear magnetic resonance, in which the mice were anesthetized with 5% chloral hydrate.

The ^19^F density-weighted ^19^F MRI phantom images of mice were acquired using the RARE sequence with the following parameters: TR = 2.5 s, TE = 4.6 ms, RARE factor = 8, number of averages = 130, number of repetitions = 1, matrix size = 48 × 48, FOV = 50 mm × 40 mm, number of slices = 1, slice thickness = 15 mm, and total acquisition time = 32.5 min. T_1_-weighted ^19^F MRI phantom images of mice were acquired using the RARE sequence with the following parameters: TR = 200 ms, TE = 4.6 ms, RARE factor = 8, number of averages = 1,630, number of repetitions = 1, matrix size = 48 × 48, FOV = 50 mm × 40 mm, number of slices = 1, slice thickness = 15 mm, and total acquisition time = 32.6 min.

*In vivo* fluorescence images were determined using an IVIS imaging system (PerkinElmer) (excitation/emission, 640/680 nm), in which the mice were anesthetized with 1%–2% isoflurane in O_2_.

#### Tracking macrophages in an inflammation mouse model

Three BALB/c mice were given footpad injections in their right paw, each containing 1% carrageenan solution (CAS# 9,064–57-7, Sigma) in 0.9% saline. After 1 h, the mice received 100 µl of E1 or E2 (C_F_ = 0.36 mmol/kg) through intravenous injection *via* the tail vein. For *in vivo* fluorescence imaging, all mice underwent serial fluorescence imaging (IVIS, PerkinElmer, America) (excitation/emission, 640/680 nm) at 2 h, 4 h, 6 h, 8 h, and 12 h after receiving E1 or E2, during which the mice were anesthetized with 1%–2% isoflurane in O_2_. A quantitative analysis of the total radiance (photons/s) was performed with Living Image software (PerkinElmer) by defining identical regions of interest covering the left (control) and right paws. *In vivo* ^19^F MR images were captured using a 9.4-T nuclear magnetic resonance, during which the mice were anesthetized with 5% chloral hydrate.

## Results and discussion

**SCHEME 1:**
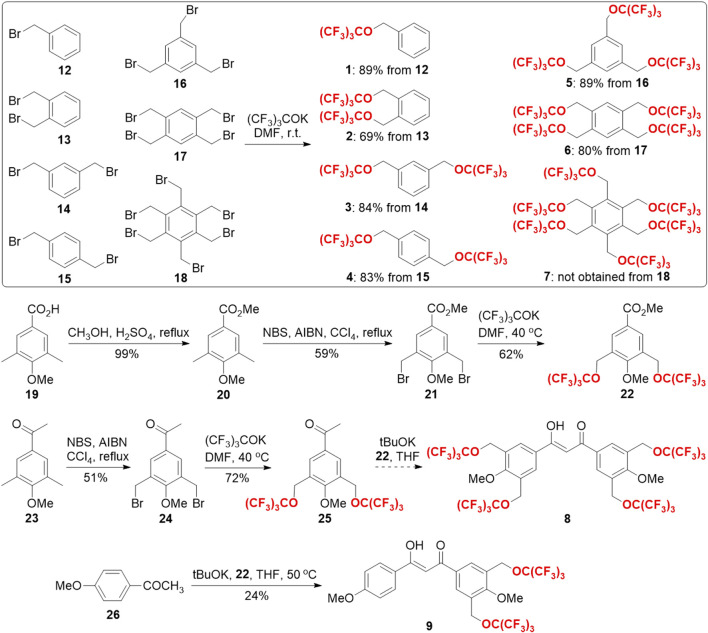
Synthesis of HFCs 1–7 and chelators 8 and 9.

With these ideas in mind, the synthesis of HFCs 1–7 and fluorinated chelators 8 and 9 was then carried out (Scheme 1). First, from a series of commercially available benzyl bromides 12–18, HFCs 1–6 were synthesized in just one step by nucleophilic substitution of the bromides with potassium perfluoro-*tert*-butoxide in *N, N*-dimethylformaldehyde (DMF). Under the optimized conditions, HFCs 1–6 were prepared in good yields on multi-gram scales. Notably, pure HFC 1 was conveniently obtained by collecting the lower phase of the water-quenched reaction mixture, and HFCs 2–6 were obtained by filtration from the mixture, followed by washing with water. Because of the high steric hindrance, many attempts failed to synthesize HFC 7 with 6 perfluoro-*tert*-butoxyl groups, which delivered complex mixtures. Second, the synthesis of fluorinated chelator 8 commenced with the construction of fluorinated ester 22 and ketone 25. The commercially available benzoic acid derivative 19 was first transformed into the corresponding methyl ester 20, which was then radically di-brominated into bromide 21. Through the potassium perfluoro-*tert*-butoxide substitution reaction mentioned earlier, fluorinated ester 22 was prepared on a gram scale with a 36% yield over three steps. Similarly, commercially available ketone 23 was di-brominated and then substituted with potassium perfluoro-*tert*-butoxide to give fluorinated ketone 25 with a 37% yield over two steps. However, the Claisen condensation between the fluorinated ester 22 and ketone 25 led to a complex mixture, probably due to the high steric hindrance and peculiar fluorous properties in the reactants. To address these issues, we replaced the fluorinated ketone 25 with commercially available 1-(4-methoxyphenyl)ethan-1-one 26 and successfully prepared partially fluorinated chelator 9 under anhydrous conditions.

**FIGURE 2:**
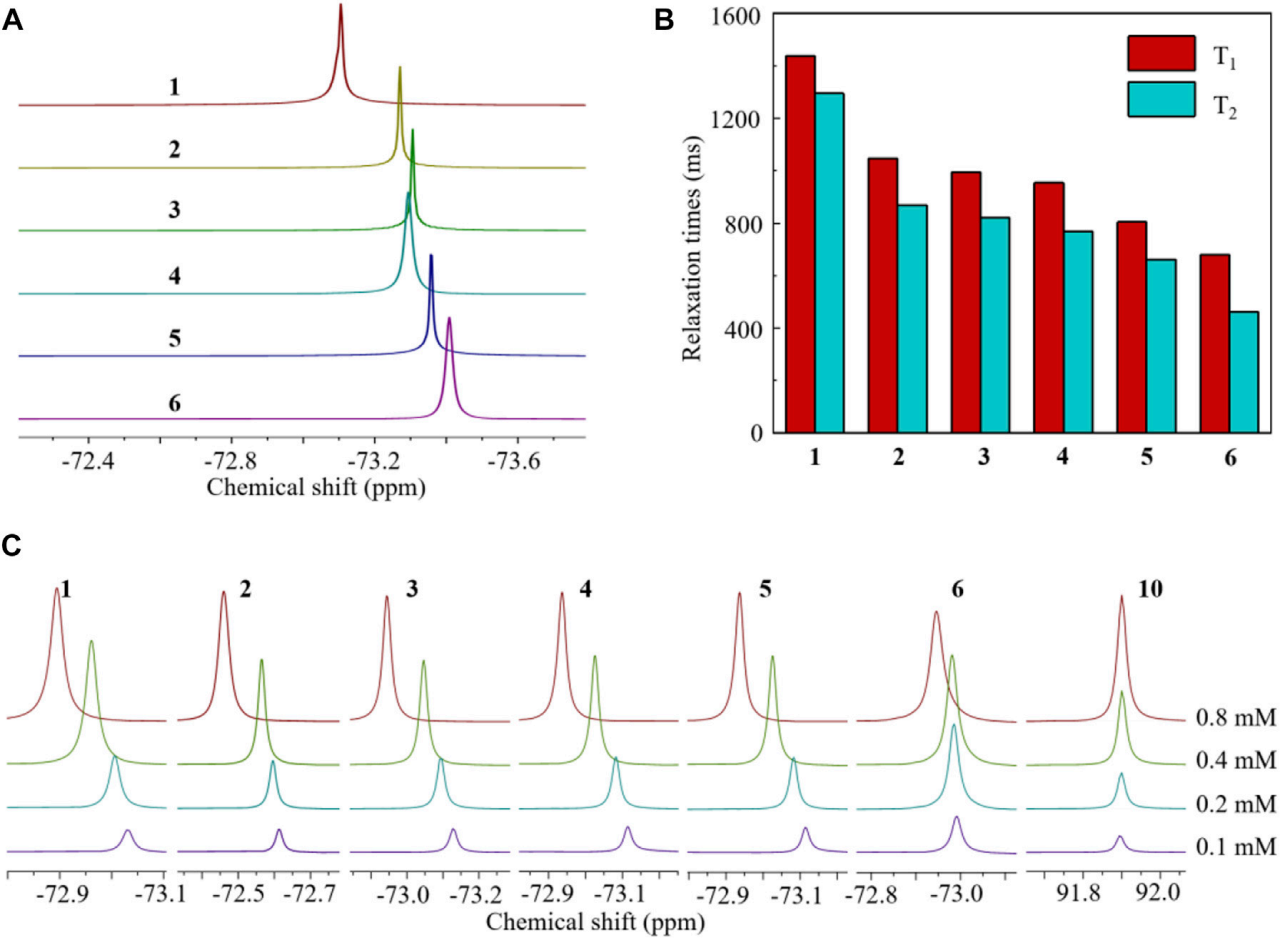
Partial ^19^F NMR spectra [**(A)**, in CDCl_3_], ^19^F relaxation times [**(B)**, 0.4 mM in PFMCH], and concentration-dependent ^19^F NMR spectra [**(C)**, in PFMCH, with perfluoro-20-crown-5 10 as a control] of HFCs 1–6. All ^19^F NMR experiments were performed at 500 MHz at room temperature. Hexafluorobene was used as the internal standard.

The structures of HFCs 1–6 and chelator 9 were confirmed by their ^1^H/^13^C/^19^F NMR and mass spectra (see Supplementary Material). The structures of known compounds 1–5 were further confirmed by comparing their ^1^H NMR spectra with those of Zhao et al. (2012). As expected, all the fluorinated compounds gave a sharp singlet ^19^F NMR peak from their multiple symmetrical fluorines, respectively (Figure 2A). HFCs 2–6 are clear solids at room temperature. Notably, solid fluorinated agents usually have an ultra-short transverse relaxation time (T_2_) and a quenched ^19^F signal. Thus, it is necessary to disperse solid HFCs 2–6 in surfactants or liquid agents to extend the T_2_ for sensitive ^19^F MRI. Interestingly, solid HFCs 2–6 showed high solubility in liquid HFC 1 due to their similar chemical structures, delivering fluorinated solutions with a pseudo singlet ^19^F NMR peak and tunable fluorine content (F%). HFCs 5 and 6, with the highest fluorine contents, showed heavy fluorous properties, for example, low solubility in most hydrocarbon solvents but high solubility in fluorous solvents like perfluoro(methylcyclohexane) (PFMCH). Thus, the ^19^F relaxation times of HFCs 1–6 were measured in PFMCH (Figure 2B). A molecular weight (MW)-dependent pattern of ^19^F relaxation time was observed from HFCs 1–6, that is, the larger the MW, the shorter the relaxation times. Compared to many perfluorocarbons, HFCs 1–6 had relatively short relaxation times and small T_1_/T_2_ ratios. During ^19^F MRI, short T_1_ and long T_2_, that is, a small T_1_/T_2_ ratio, are beneficial for rapid data collection and thus sensitive ^19^F MRI. It was noteworthy that the π–π interactions among the benzene cores were successfully observed in the concentration-dependent ^19^F NMR spectra of HFCs 1–6 in PFMCH (Figure 2C). When increasing the concentrations, the ^19^F NMR peaks of HFCs 1–6 were shifted to the low field as far as 0.184 ppm in HFC 3. In contrast, neglectable apparent chemical shift changes, about 0.005 ppm, were observed in the corresponding solutions of perfluoro-20-crown-5 10, which has no benzene structure. Furthermore, HFC 6 with four bulky PFTB groups gave a minimal ^19^F chemical shift change of 0.045 ppm among the HFCs, in which the high steric hindrance severely reduced the π–π interactions. In pure HFC 1 and its HFC 2–6 solutions, the π–π interactions were supposed to reduce the T_1_ for sensitive ^19^F MRI. Therefore, HFCs 1–6 were conveniently synthesized on relatively large scales as valuable and sensitive ^19^F MRI agents with a high F%, a sharp singlet ^19^F NMR peak, significant π–π interactions among the benzene cores, and relatively short relaxation times.

Because of its liquid form, very cheap precursor, and convenient multigram-scale synthesis, HFC 1 was selected to formulate partially fluorinated nanoemulsions for ^19^F MRI cell tracking. Due to their similar chemical structures, fluorinated chelator 9 was quite soluble in HFC 1, dramatically simplifying the formulation of paramagnetic nanoemulsions. S75 and egg yolk lecithin together with F68 were, respectively, identified as the optimal surfactants for the formulation, providing nanoemulsions of HFC 1, chelator 9, and aza-BODIPY 11 using a thin-film dispersion method (Table 1, Entries 1 and 2). Interestingly, adding a small amount of soybean oil delivered highly monodisperse nanoemulsions E1 and E3 (Table 1, Entries 3 and 5). After adding FeCl_3_ to the nanoemulsion solution, the apparent color change indicated the chelation of Fe^3+^ by fluorinated chelator 9, providing a paramagnetic nanoemulsion with negligible particle size and PDI changes. After the removal of unchelated Fe^3+^ in the solutions by dialysis, paramagnetic nanoemulsions E2 and E4 were prepared with high monodispersity (Table 1, Entries 4 and 6).

**TABLE 1:** Ingredients, particle sizes, and PDIs of partially fluorinated nanoemulsions.

Entry	Ingredients^a^	Size^b^ (PDI)
1	1, 9, 11, and S75	127 (0.23)
2	1, 9, 11, lecithin, and F68	113 (0.32)
3	1, 9, 11, S75, and soybean oil (E1)	143 (0.14)
4	1, 9, 11, S75, soybean oil, and FeCl_3_ (E2)	145 (0.14)
5	1, 9, 11, lecithin, F68, and soybean oil (E3)	189 (0.19)
6	1, 9, 11, lecithin, F68, soybean oil, and FeCl_3_ (E4)	189 (0.21)

^a^ Amount of ingredients in 4 ml water: 130 mg 1, 2 mg 9, 1 mg 11, and 10 mg soybean oil.

^b^ Particle sizes were measured by DLS, as nm of the diameter.

**FIGURE 3:**
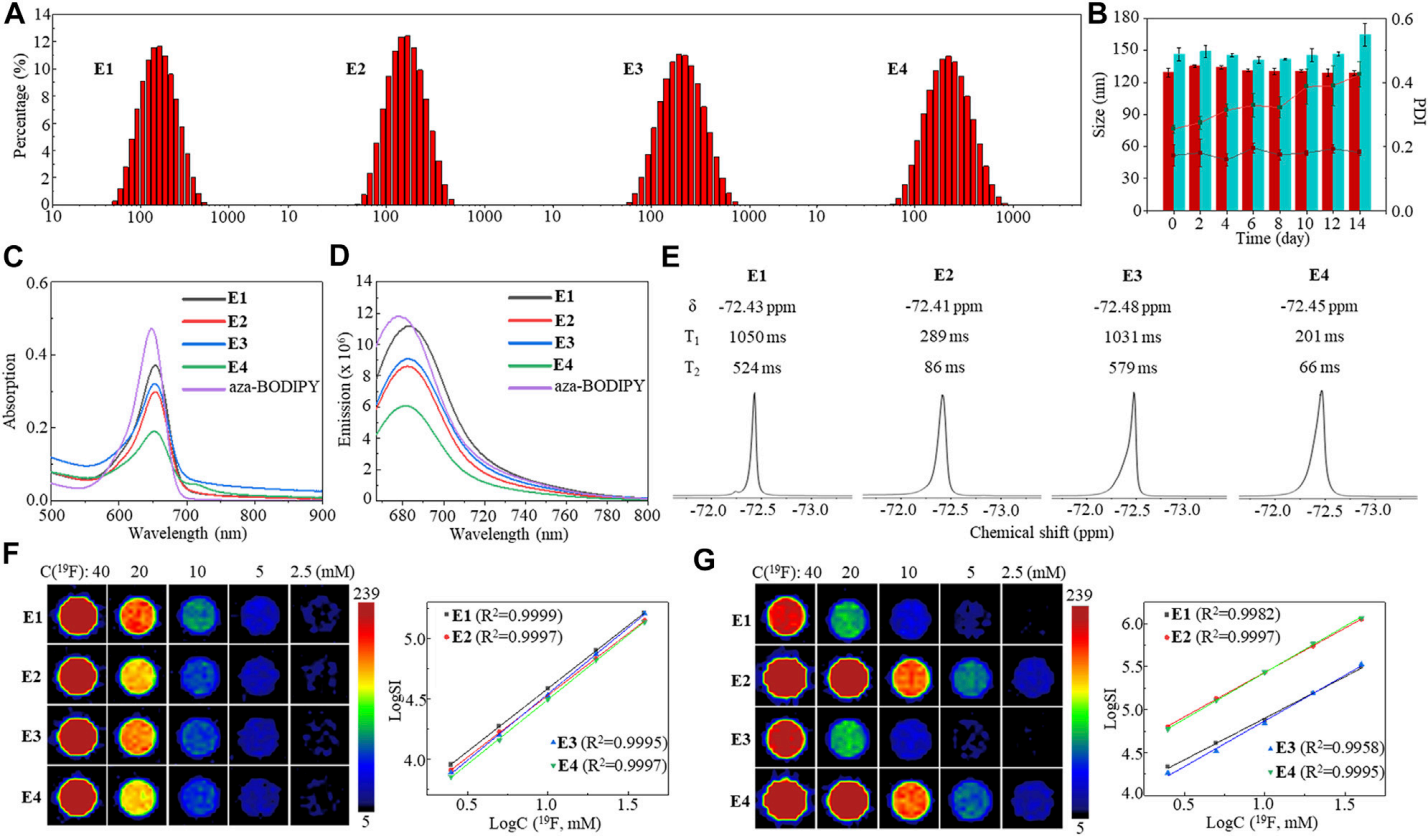
DLS **(A)**, stability monitored by DLS over 14 days **(B)**, UV-Vis absorption spectra **(C)**, FL emission spectra **(D)**, partial ^19^F NMR spectra and ^19^F relaxation times **(E)**, ^19^F density-dependent phantom images and the logarithm plot of SI versus ^19^F concentration **(F)**, and ^19^F T_1_-dependent phantom images and the logarithm plot of SI versus ^19^F concentration **(G)** of nanoemulsions E1–E4.

The high monodispersity of nanoemulsions E1–E4 was confirmed by dynamic light scattering (DLS) with low polydispersity indexes (PDI, Figure 3A). Furthermore, DLS detected only slight changes in the particle size and PDI of E1 solutions during 14 days (Figure 3B), indicating its high stability. In contrast, a considerable increase in the PDI of E3 between day 8 and day 14 showed less efficacy of lecithin and F68 in formulating HFC 1. NIR dye aza-BODIPY 11 was successfully encapsulated into the nanoemulsions with high efficiency (E1: 93%, E2: 96%, E3: 93%, and E4: 97%) and a loading content of about 0.45%. Compared to aza-BODIPY 11, the nanoemulsions gave slightly red-shifted maximum UV-Vis absorption peaks around 653 nm (Figure 3C) and a maximum FL emission peak around 682 nm (Figure 3D). Moreover, the encapsulation did not compromise the fluorescence intensity of aza-BODIPY (Supplementary Figure S3). It was found that the Fe^3+^ in nanoemulsions E2 and E4 considerably lowered the maximum UV-Vis absorption and FL emission intensities, probably due to the photoinduced electron transfer between aza-BODIPY and Fe^3+^ (El-Khouly et al., 2004; Doose et al., 2009; Fukuzumi et al., 2012). Each fluorinated nanoemulsion gave a singlet ^19^F NMR peak of around −72.4 ppm (Figure 3E). The nanoemulsions E1 and E3 had relatively long ^19^F T_1_, while the Fe^3+^ in nanoemulsions E2 and E4 significantly shortened the ^19^F T_1_ by 3.6 and 5.1 folds, respectively, through the PRE effect (Figure 3E). The short T_1_ facilitated a fast ^19^F T_1_-dependent ^19^F MRI scan for high-quality images. The nanoemulsions E1–E4 were imaged by ^19^F MRI at a ^19^F concentration as low as 5 mM using a ^19^F density-dependent MRI method with a data collection time of 384 s (Figure 3F). As expected, the ^19^F MRI sensitivity was significantly improved by the encapsulated Fe^3+^ using a ^19^F T_1_-dependent MRI method, in which paramagnetic nanoemulsions were imaged at a low concentration of 2.5 mM with a data collection time of 389 s (Figure 3G). Notably, the logarithm of ^19^F signal intensity (SI) was proportional to the ^19^F concentration in both density-dependent ^19^F MRI and T_1_-dependent ^19^F MRI (Figures 3F, G), which facilitates the quantification of ^19^F concentration with ^19^F SI. Thus, fluorinated nanoemulsions E1–E4 with high monodispersity, stability, and unified and paramagnetic-enhanced ^19^F signals were formulated as sensitive and quantitative ^19^F MRI-FLI dual imaging agents.

**FIGURE 4:**
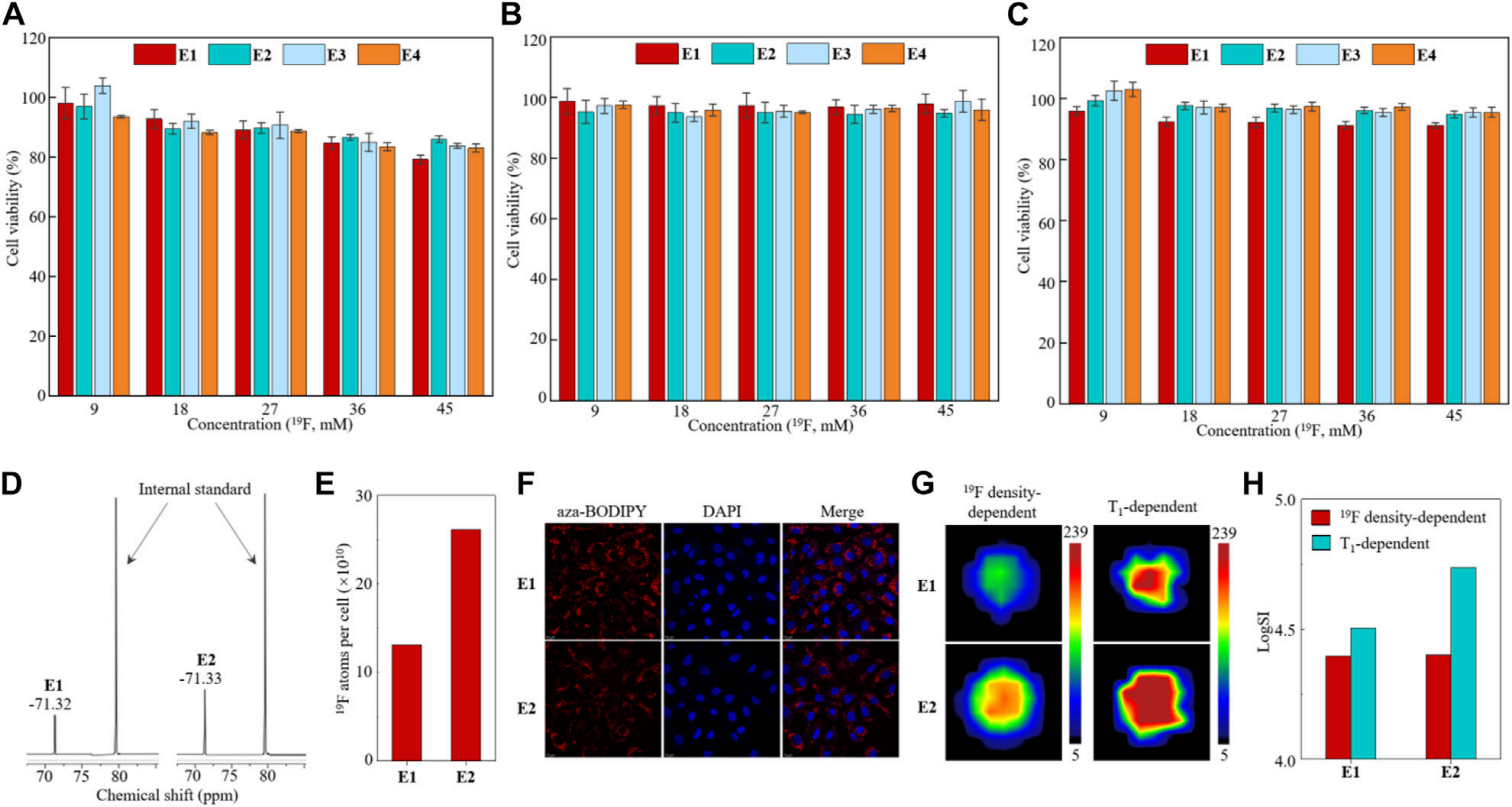
Cytotoxicity assay of nanoemulsions E1–E4 in A549 cells **(A)**, MCF-7 cells **(B)**, and MDA-MB-231 cells **(C)**. Partial ^19^F NMR spectra **(D)** and quantitative analysis of ^19^F atoms’ uptake in each cell **(E)** of nanoemulsion E1- and E2-treated A549 cell lysates. Confocal microscopy images of A549 cells treated with nanoemulsion E1 and E2 after 12 h of incubation **(F)**. ^19^F MRI images **(G)** and quantitative analysis of ^19^F SI **(H)** of nanoemulsion E1- and E2-treated A549 cells.

Next, nanoemulsions E1–E4 were investigated in cells. First, the biocompatibility of nanoemulsions E1–E4 was investigated in human lung cancer A549 cells, human breast cancer MCF-7 cells, and human triple-negative breast cancer MDA-MB-231 cells using the CCK-8 cytotoxicity assay. High cell viabilities were observed in the nanoemulsion-treated cells with a ^19^F dose as high as 45 mM after 12 h of incubation (Figures 4A–C), far beyond the ^19^F MRI-detectable concentrations mentioned earlier. Second, due to their relatively high stability, a quantitative ^19^F NMR study was carried out on the nanoemulsion E1- and E2-treated A549 cells. After incubating 1 × 10^7^ A549 cells with nanoemulsions E1 and E2 at a ^19^F concentration of 18 mM for 12 h, the cell lysates were quantitatively analyzed with ^19^F NMR, which gave a strong ^19^F NMR peak of around −71.3 ppm, respectively (Figure 4D). After calibrating the ^19^F NMR SI with an internal standard of sodium triflate, each cell was found to take up 10^11^ fluorine atoms (Figure 4E). Interestingly, A549 cell lines showed significantly higher uptake of paramagnetic nanoemulsion E2 than diamagnetic nanoemulsion E1, while the reason for this phenomenon is still unclear. Third, the cell uptakes of nanoemulsions E1 and E2 were studied with confocal microscopy in A549 cells (Figure 4F). After 12 h of incubation, the red fluorescence of aza-BODIPY was visible in the cytoplasm around the nucleus dyed with blue-fluorescent DAPI, indicating the successful uptake of nanoemulsions E1 and E2. Notably, the Fe^3+^ partially quenched the FL of aza-BODIPY in nanoemulsion E2, resulting in a weaker FL intensity of E2-treated cells than that of E1-treated cells. In addition, no notable hemolysis was observed for nanoemulsions E1 and E2 in the blood (Supplementary Figure S2), and no death of BALB/c mice was observed after intravenous injection of nanoemulsion (100 μl, ^19^F concentration of 450 mM) in an acute toxicity test, indicating the good biocompatibility of nanoemulsions E1 and E2. Finally, ^19^F MRI was carried out on the E1- and E2-treated A549 cells. About 1 × 10^7^ A549 cells were clearly imaged by both ^19^F density-dependent and T_1_-dependent ^19^F MRI with short data collection times of 384 s and 389 s, respectively (Figure 4G). Moreover, due to the PRE effect of Fe^3+^, the SI of T_1_-dependent ^19^F MRI was 2.2-fold higher than that of ^19^F density-dependent ^19^F MRI in the mice injected with paramagnetic nanoemulsion E2-treated A549 cells (Figure 4H). Therefore, the partially fluorinated nanoemulsions E1 and E2 had high biocompatibility, quantifiable ^19^F NMR, efficient cell uptake, and high ^19^F MRI sensitivity, which were suitable for quantitative ^19^F MRI-FLI dual imaging cell tracking.

**FIGURE 5:**
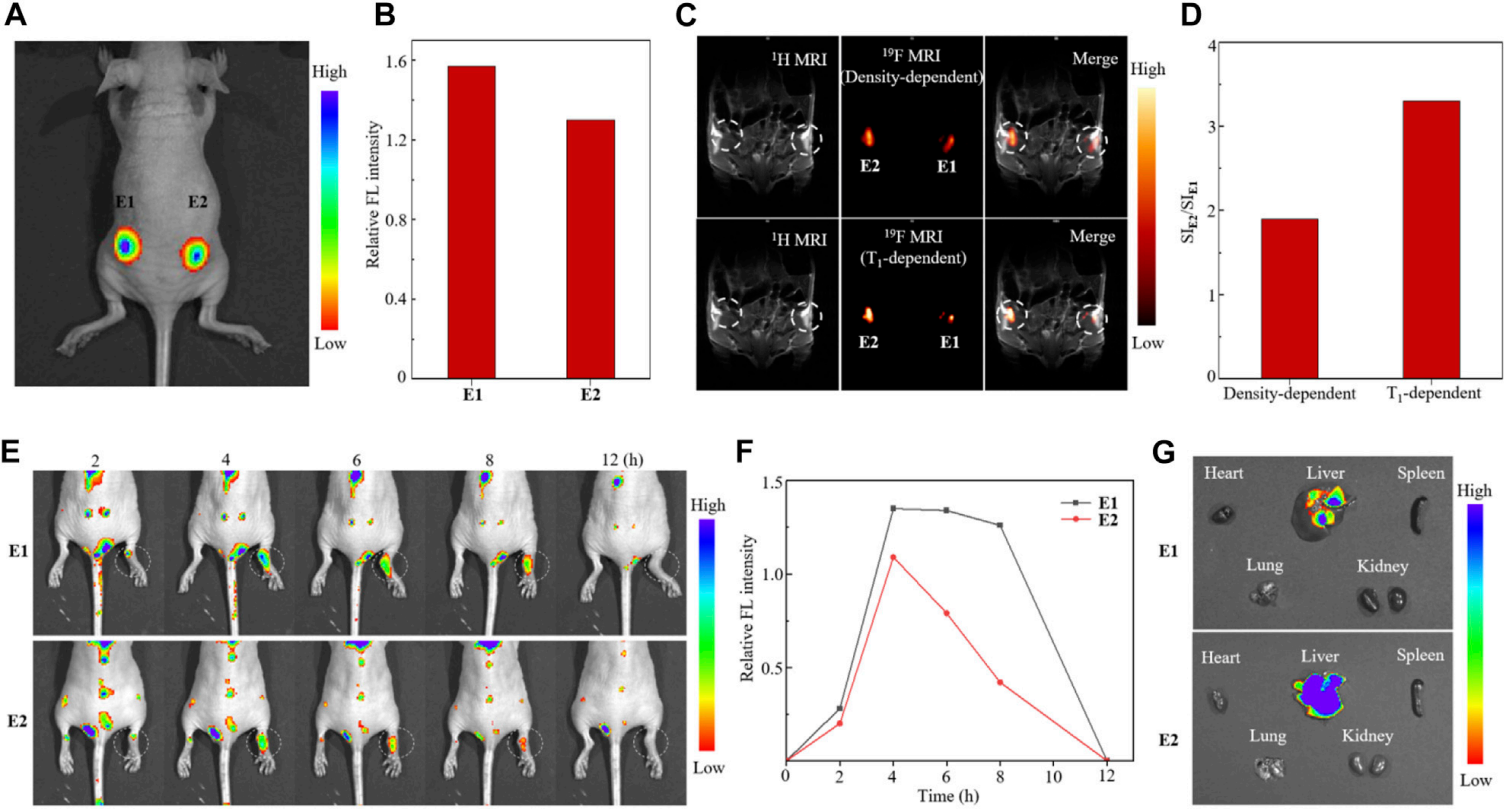
Fluorescence image **(A)**, quantitative analysis of relative FL intensity **(B)**, ^19^F MRI [**(C)**, upper: ^19^F density-dependent images, lower: ^19^F T_1_-dependent images], and quantitative analysis ^19^F SI **(D)** of nude mice injected with nanoemulsion E1- and E2-labeled A549 cells. Time-dependent FL images **(E)**, quantitative analysis of FL intensity in the right paw region **(F)**, and FL images of internal organs [**(G)**, collected 4 h post-injection] of mice with carrageenan-induced paw inflammation after tail vein injection of nanoemulsions E1 and E2.

Finally, *in vivo* ^19^F MRI-FLI dual-modal cell tracking with partially fluorinated nanoemulsions E1 and E2 was carried out in BALB/c nude mice. On the one hand, the tracking of lung cancer A549 cells was investigated by subcutaneous injection of 1 × 10^7^ E1- and E2-labeled A549 cells to the right and left flanks of mice, respectively (Figure 5A). The injected cells were first tracked with *in vivo* FL, in which paramagnetic nanoemulsion E2-treated A549 cells showed weaker FL intensity than that of E1-treated cells due to the FL-quenching Fe^3+^ in nanoemulsion E2 (Figure 5B). Then, the A549 cells were tracked with ^19^F MRI using ^19^F density-dependent and T_1_-dependent MRI sequences (Figure 5C). Notably, the cells injected into the left flank gave a 1.9-fold higher ^19^F MRI SI than that of the right flank using a ^19^F density-dependent sequence, while the ratio of SI was further improved to 3.3-fold using a T_1_-dependent sequence (Figure 5D), showing the high efficacy of Fe^3+^ in improving ^19^F MRI sensitivity. On the other hand, macrophage cells were labeled *in vivo* and tracked in a carrageenan-induced inflammation model. After tail vein injection of 100 μl nanoemulsions E1 and E2 at a ^19^F concentration of 72 mM into the mice with right paw inflammation, the time-dependent FLI and ^19^F MRI were recorded, respectively. The time-dependent FL images showed that the nanoemulsion-labeled macrophages accumulated mainly in the liver, lung, and kidney at 2 h post-injection and selectively accumulated in the right paw at 4 h post-injection, while neglectable FL was observed in the left paw (Figure 5E). The time-dependent FL intensity in the right paw region indicated that E2-labeled macrophages were eliminated much faster than E1-labeled macrophages in this inflammation model (Figure 5F). The FL images of internal organs collected at 4 h post-injection illustrated the nanoemulsion mainly accumulated in the liver, while the accumulations in the heart, spleen, lung, and kidneys were not detected at this time (Figure 5G). Unfortunately, many attempts to track the nanoemulsion-labeled macrophages with ^19^F MRI could not provide clear images of the inflamed paw, probably due to the low dose of E1 and E2.

## Conclusion

In summary, we have developed partially fluorinated paramagnetic nanoemulsions from readily available chemicals for ^19^F MRI-FLI dual-modal cell tracking. To address the challenge of ^19^F MRI sensitivity in cell tracking, we have used many valuable strategies, including the efficient synthesis of fluorinated molecules with multiple symmetrical fluorine atoms and a strong ^19^F signal, unifying the ^19^F signals from all components in the nanoemulsions and shortening T_1_ through the intermolecular interactions and the PRE effect of paramagnetic iron (III). Indeed, the partially fluorinated paramagnetic nanoemulsions exhibit high ^19^F MRI sensitivity, which was detected at a low fluorine concentration of 2.5 mM with a short data collection time of 389 s. Further improvement in ^19^F MRI sensitivity, for example, using HFCs 5 and 6 as ^19^F signal molecules, is still needed to achieve more sensitive *in vivo* ^19^F MRI cell tracking as indicated in the inflammation mice model. Meanwhile, efforts have been made to improve partially fluorinated nanoemulsions’ physicochemical and biological properties. Through screening surfactants and adding soybean oil, highly monodispersed and stable nanoemulsions were obtained. The significant intra-molecular interactions of perfluoro-*tert*-butyl benzyl ethers have been shown to facilitate the successful encapsulation of functional molecules, including fluorinated chelators and aza-BODIPY, into stable nanoemulsions, which enables their dual-modal imaging and high biocompatibility toward a series of cells. Because of their peculiar physicochemical properties, the application of perfluorocarbon nanoemulsions in ^19^F MRI cell tracking has been hampered by issues of sensitivity, biocompatibility, and multimodal compatibility. The strategy developed here, lowering the fluorine content while maintaining sensitivity and optimizing physicochemical properties, may address some of these issues and push ^19^F MRI cell tracking into further clinical application.

## Data Availability

The original contributions presented in the study are included in the article/Supplementary Material; further inquiries can be directed to the corresponding authors.
