# Supplementary material for: 19F MRI-fluorescence imaging dual-modal cell tracking with partially fluorinated nanoemulsions
Source: Front Bioeng Biotechnol. 2022 Nov 3;10:1049750. doi: 10.3389/fbioe.2022.1049750 (PMC9669590; doi:10.3389/fbioe.2022.1049750)
Supplement: Supplementary file 1 [file DataSheet1.DOCX]

Supplementary Material

**1 Supplementary Figures**

**
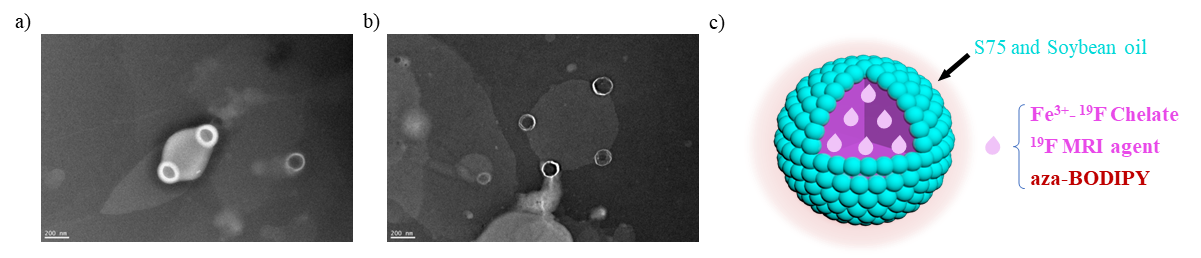
**

**Figure S1.** TEM images of nanoemulsions **E1** (a) and **E2** (b), and the structure of nanoemulsions (c).

**
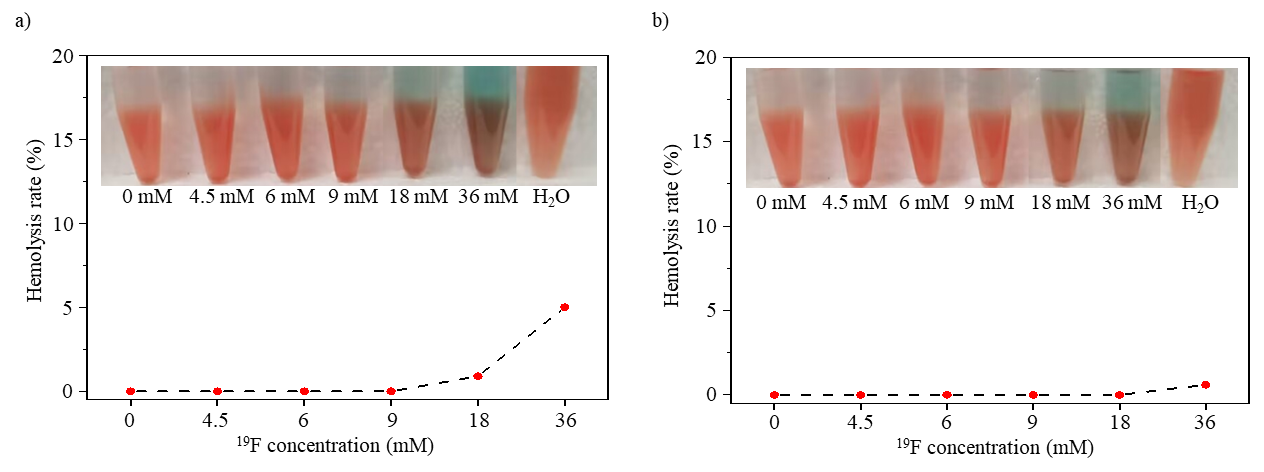
**

**Figure S2.** Hemocompatibility test of nanoemulsions **E1** (a) and **E2** (b), deionized water and PBS were used as positive and negative controls, respectively.


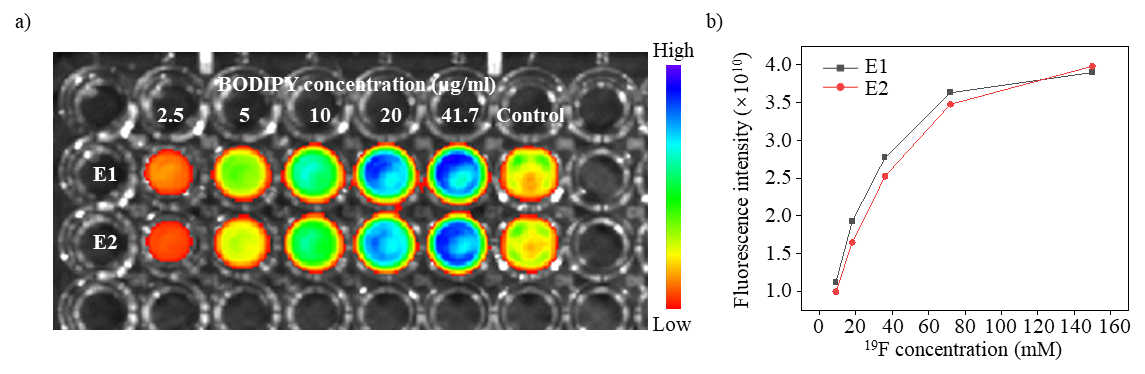


**Figure S3.** *In vitro* fluorescence images (a) and quantitative analysis of FL intensity (b) of nanoemulsions **E1** and **E2** at different concentrations. The concentration of aza-BODIPY in the control group is 2.5 µg/mL.


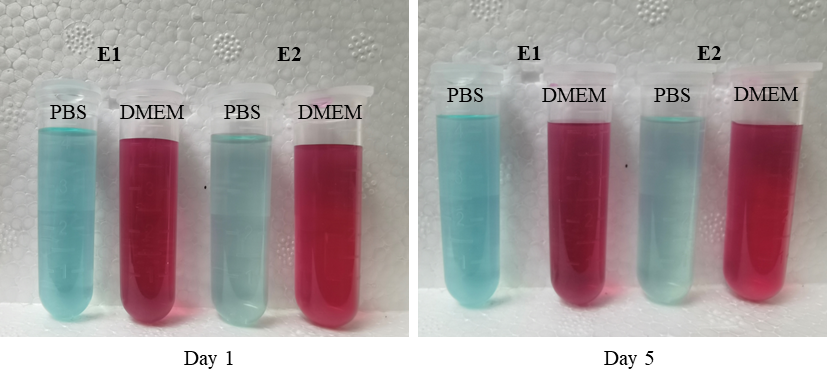


**Figure S4.** The digital photographs of nanoemulsions **E1** and **E2** in PBS and DMEM for 5 days.

**2 Copies of ^1^H/^13^C/^19^F NMR and HRMS Spectra of Compounds**

^1^H NMR of compound **1**

^19^F NMR of compound **1**

^1^H NMR of compound **2**

^19^F NMR of compound **2**

^1^H NMR of compound **3**

^19^F NMR of compound **3**

^1^H NMR of compound **4**

^19^F NMR of compound **4**

^1^H NMR of compound **5**

^19^F NMR of compound **5**

^1^H NMR of compound **6**

^19^F NMR of compound **6**

^13^C NMR of compound **6**

HRMS of compound **6**

^1^H NMR of compound **9**

^19^F NMR of compound **9**

^13^C NMR of compound **9**

HRMS of compound **9**

^1^H NMR of compound **20**

^1^H NMR of compound **21**

^13^C NMR of compound **21**

HRMS of compound **21**

^1^H NMR of compound **22**

^19^F NMR of compound **22**

^13^C NMR of compound **22**

HRMS of compound **23**

^^

^1^H NMR of compound **24**

^13^C NMR of compound **24**

HRMS of compound **24**

^1^H NMR of compound **25**

^19^F NMR of compound **25**

^13^C NMR of compound **25**

HRMS of compound **25**
